# Supplementary material for: Splice-Junction-Based Mapping of Alternative Isoforms in the Human Proteome
Source: Cell Rep. Author manuscript; Available in PMC 2020 Jan 15. (PMC6961840; doi:10.1016/j.celrep.2019.11.026)
Supplement: 2 [file NIHMS1546469-supplement-2.zip › DF1/PXD010154/Testis/Testis_45_KIF21B_QLALGNVISALGDQSK.pdf]

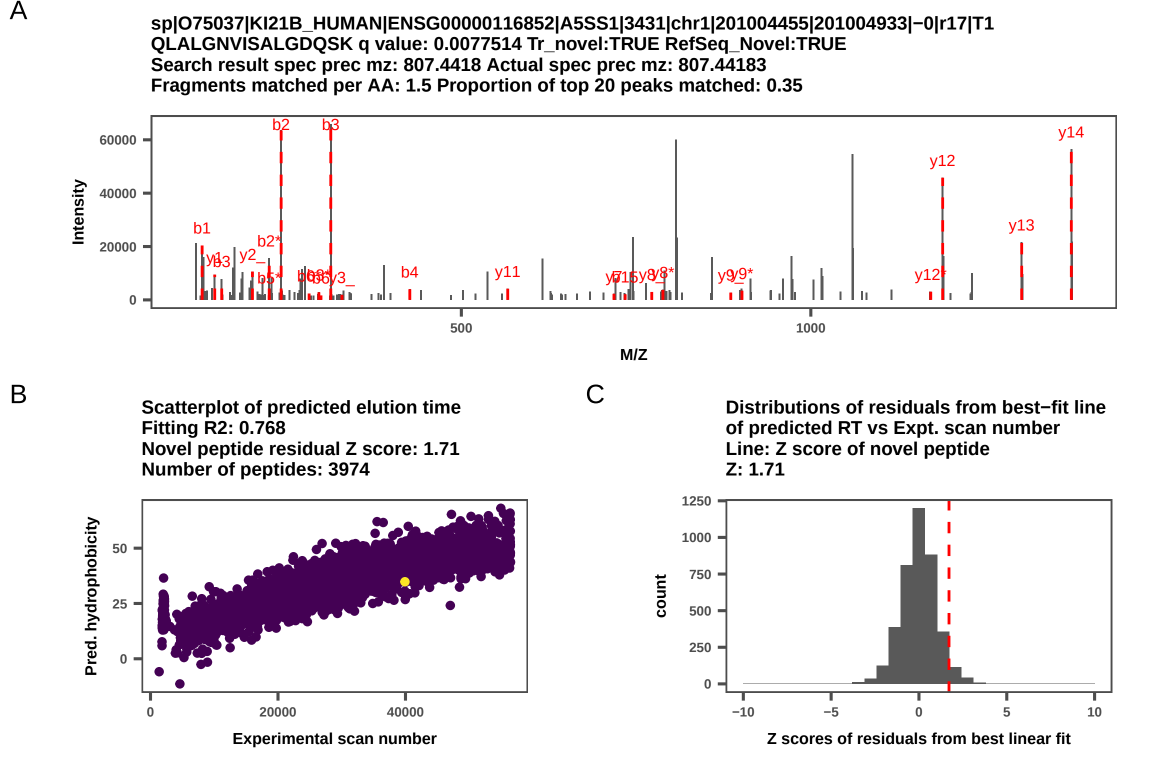
A
sp|O75037|KI21B_HUMAN|ENSG00000116852|A5SS1|3431|chr1|201004455|201004933|−0|r17|T1
QLALGNVISALGDQSK q value: 0.0077514 Tr_novel:TRUE RefSeq_Novel:TRUE
Search result spec prec mz: 807.4418 Actual spec prec mz: 807.44183
Fragments matched per AA: 1.5 Proportion of top 20 peaks matched: 0.35
Intensity
60000
40000
20000
0
b1
b2
b3
b2*
b4
y11
y12
y12*
y13
y14
500
1000
M/Z
B
Scatterplot of predicted elution time
Fitting R2: 0.768
Novel peptide residual Z score: 1.71
Number of peptides: 3974
Pred. hydrophobicity
50
25
0
0
20000
40000
Experimental scan number
C
Distributions of residuals from best−fit line
of predicted RT vs Expt. scan number
Line: Z score of novel peptide
Z: 1.71
count
1250
1000
750
500
250
0
−10
−5
0
5
10
Z scores of residuals from best linear fit
